# Implementation and Evaluation of an Emergency Department-Based Procedure Task Trainer Cart: A Mixed Methods Study

**DOI:** 10.7759/cureus.107363

**Published:** 2026-04-19

**Authors:** Kathryn Oskar, Nicole Danaher-Garcia, Reem J Alansari, Giovanni Rodriguez, Janice C Palaganas

**Affiliations:** 1 Emergency Medicine, University of Vermont, Burlington, USA; 2 Biostatistics, School of Healthcare Leadership, MGH Institute of Health Professions, Boston, USA; 3 Medical Education, Arabian Gulf University, Manama, BHR; 4 Emergency Medicine, Brigham and Women's Hospital, Harvard Medical School, Boston, USA; 5 Health Professions Education, MGH Institute of Health Professions, Boston, USA

**Keywords:** emergency medicine resident, emergency procedure, procedure training, simulation-based education, task trainer

## Abstract

Introduction

Teaching procedural skills in the emergency department (ED) can be challenging. To support on-shift procedural training in a busy adult academic ED setting, a dedicated modular cart containing simulation-based procedure task trainers and supplies was created and placed into the clinical space.

Methods

Uptake among ED providers was assessed via the number of logged cart accesses over time. The educational impact of increased availability of simulation equipment on shift was assessed using pre- and post-intervention surveys of cart users and semi-structured interviews with resident physician cart users.

Results

Use of the trainer cart was initially robust at 13 uses per month, but decreased significantly to one to two uses per month after the initial pilot phase. Pre- and post-survey data, while underpowered, indicated trends in on-shift training time and a preference for using equipment with near peers rather than for solo practice. Thematic analysis of semi-structured interviews revealed the following three themes around the perceived utility of task trainers: (1) “practice makes perfect,” (2) “applications beyond the tool,” and (3) “limitations, barriers, and solutions.”

Conclusion

Making simulation-based procedure training more conveniently accessible and closer to real-world practice by placing a task trainer cart in the clinical setting was well-received by clinicians when it was used. Interviews offered rich insights into specific improvements for future iterations of the cart and added understanding of how similar interventions may fit into the longitudinal learning curve of procedural skill acquisition and maintenance among emergency medicine trainees.

## Introduction

Effectively teaching procedural skills in the emergency department (ED) can be challenging. Building procedural competence in emergency medicine (EM) trainees is essential for long-term patient safety and outcomes, yet the cognitive demands and rapid pace of the clinical environment, unpredictable, irregular, and sometimes infrequent opportunities for procedure practice, and the instability of patients requiring emergent procedures are often barriers to safe and effective procedural teaching and learning on shift. Simulation-based training is a widely accepted and widely used mechanism for learners to refine their skills, improve competence through deliberate practice, and increase knowledge and confidence in a safe environment [1-5]. However, simulation-based training often occurs in the simulation lab during dedicated low-stakes training sessions. This raises questions about whether skills may not entirely transfer from the lab to the patient, or whether skills may decay over long periods between simulation lab practice and a clinical encounter. To enhance EM resident procedural training on shift, an easily accessible simulation-based task trainer cart was created. The envisioned use was that the cart supplies were to support “just-in-time” (JIT) training, or anticipatory simulation, a type of training conducted just prior to an anticipated clinical event to improve real-world performance [6]. JIT teaching has been shown to improve procedural performance in a spectrum of clinical settings, and specifically among EM physicians [7,8]. While a fair amount has been published on the use of video and other digital technologies to support JIT training in EM, and prior studies of similar task trainer interventions in a pediatric ED setting showed improved confidence and skills [9,10], few studies have evaluated how task trainers may be utilized for JIT or other types of simulation-based education in an adult ED setting [5].

## Materials and methods

The intervention took place in a large urban ED that houses a four-year emergency medicine (EM) residency training program. Institutional review board approval was obtained prior to the introduction of the cart and the initiation of data collection (MGB IRB #2024P001258).

Description of the training cart

**Figure 1:**
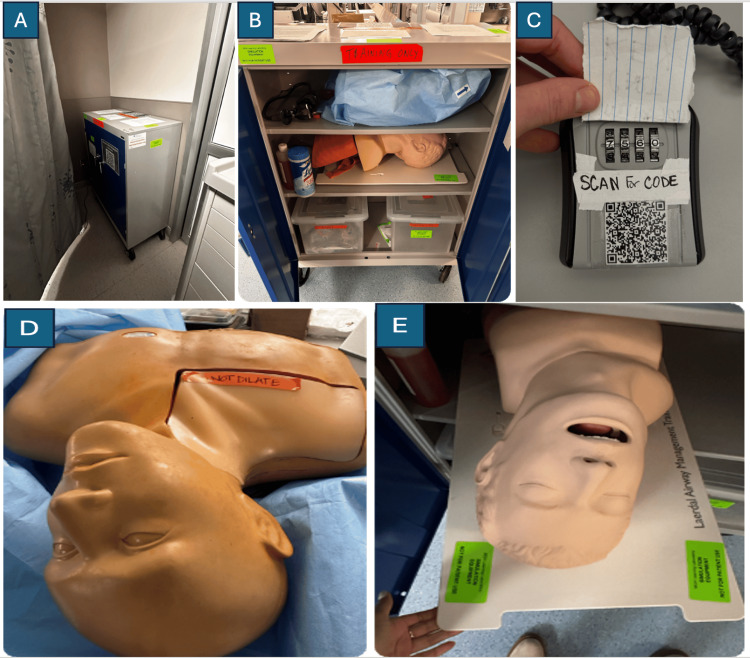
Simulation task trainer cart. (A) Cart locked and housed in ED bay. (B) Open task trainer cart showing trainers and additional procedure supplies. (C) The key to open the trainer cart was stored in a lockbox at the attending physician's workstation. Lockbox opened with a four-digit code provided after access was logged via a QR-code accessible form. (D) CVC trainer housed in the cart. (E) Airway trainer is housed in the trainer cart. CVC: central venous catheter

A lockable task trainer cart was housed in the ED (Figures 1A-1E). The cabinet and all supplies were lent from the institution’s simulation lab at no cost to the authors. To access the key to unlock the trainer cart, individuals were required to log their use by scanning a QR code that linked to a REDCap version 15.0.34, 2025 (Nashville, TN: Vanderbilt University) [11]. All trainers, supplies, and the cabinet itself were clearly labeled “for simulation purposes only, not for clinical use” to align with best practices for safety during in situ simulation [12]. Simulation trainers placed in the cart were the Laerdal Airway Management Trainer (Stavanger, Norway: Laerdal Medical Corporation) and the CAE Blue Phantom Gen II Ultrasound Central Line Training Model (Sarasota, FL: CAE Healthcare). The task trainers, manufacturer-provided trainer user guides, and a variety of all necessary supplies for central line placement and endotracheal intubation were placed in the cart. Additionally, mastery-learning checklists for orotracheal intubation and internal jugular central venous catheter placement, along with a cell phone holder device to facilitate participant self-recording for video review on personal devices, were placed in the cart for optional use [13]. Endotracheal intubation and central venous cannulation were selected as the first procedures for the task trainer cart because they are required for EM residency graduation by the Accreditation Council for Graduate Medical Education (ACGME), and thus core to the practice of EM, and occur commonly enough that the authors suspected JIT practice could feasibly occur during the pilot phase.

The training equipment could be accessed as often as desired and used at the discretion of the individuals accessing the supplies. There was no prerequisite training, knowledge, or other requirement. The anticipated use of the trainers was for just-in-time (JIT) procedure practice by junior learners with coaching from a supervising clinician to optimize procedural success before performing an anticipated procedure on a patient. However, individuals, including attending physicians, resident physicians, and physician assistants, could access the cart and use the trainers in other ways to meet user-centered educational needs. Individuals who accessed the simulator cart were responsible for the cleaning and return of the equipment. The cart and equipment were assessed monthly while in the ED by two of the study’s authors to ensure their upkeep.

Pilot program

The introduction of the training cart, including location, supplies, and anticipated uses, was announced at EM faculty and resident conferences. For the first three months of the trainer cart being placed in the clinical environment, observational data were collected to assess the adoption rate and user demographics. Users were required to log each time the cart was accessed via a QR-linked REDCap form, as described above. This form asked participants to provide demographic information, including level of training and whether they were using materials to teach others or as a learner. Cart use was voluntary by clinicians working in the ED and was not limited to any specific role group during the pilot phase. The total logged uses were used to quantify the adoption rate of the procedure trainer cart. Descriptive user data gave a preliminary idea of how clinicians were using the cart. Informed consent to provide demographic and contact information was obtained from participants via the REDCap form, and records of logged use were maintained in an institution-secured, password-protected database.

Pre- and post-survey

After successfully piloting the trainer cart, pre- and post-use surveys were developed to better assess the educational impact of the cart. Confidence when performing central venous catheter placement and endotracheal intubation before and after using the cart was rated by the participant using a five-point Likert scale (1=not confident at all to 5=very confident). Surveys were created using REDCap version 15.0.34 (2025) and developed through a review of the existing literature and in collaboration with two experts in medical education research methodology. Informed consent was explicitly obtained prior to participation in surveys. Self-reported competence was not assessed due to prior studies indicating the limited accuracy of self-assessment in procedural skills [14]. Pre- and post-survey responses were planned for analysis using paired t-tests; however, given the small number of complete pre- and post-responses, these analyses could not be performed (appendix 1).

An invitation to participate in the pre-survey was added to the same QR code-accessible REDCap form that participants used to log in and gain access to the cart. After completion of the pre-survey, a link to the post-survey was immediately emailed to the participant so that the post-survey could be completed immediately or shortly after working with the intervention. If the post-survey was not completed within two days of pre-survey completion, a reminder email with a link to the post-survey was sent automatically via REDCap.

Physician user interviews

EM resident physicians who had logged use of the task trainer cart at any time between March 2024 and March 2025 and had consented to providing their personal data to this study were sent email invitations to participate in a 15-min semi-structured interview with one of the primary authors of the study (appendix 2). Interviews were conducted via the institution-supported Zoom version 6.5.9 (San Jose, CA: Zoom Communications, Inc.) and were audio- and video-recorded. Interviews were transcribed using Zoom, and transcripts were reviewed to confirm accuracy and completeness of transcription. Transcripts were then uploaded to Dedoose version 9.2.22, 2025 (Los Angeles, CA: SocioCultural Research Consultants, LLC), a software that allows for coding and thematic analysis. Interviews were continued until saturation was reached, at which point no new codes were identified [15]. Seven interviews were completed.

Thematic analysis was completed using Braun and Clarke’s six-step framework [16]. After initial reading through transcripts to gain familiarity with the data, codes were generated by the authors independently. Next, consensus on the final codes was reached through discussion among the authors, and the dataset of interview transcripts was then reread and recoded using the established and agreed-upon codes in Dedoose. Using Miro version 3.6.1 (2025) (San Francisco, CA: RealtimeBoard) concept-mapping software, codes were combined into themes. These themes were again agreed upon by discussion and consensus among the authors. Themes were reviewed in relation to coded extracts and to the entire data set to confirm that themes adequately captured the interview participants’ responses.

## Results

Adoption rate

Initial results of cart usage during the pilot phase from March 1, 2024, to May 1, 2024, are represented in Table 1. Pilot data demonstrated that the task trainer cart was used by 21 unique individuals, all of whom were EM residents. Given 60 total residents in the program, this represented about one-third of EM residents who used the training supplies. The cart was accessed a total of 26 times during the pilot phase, an average of 13 uses per month. No adverse events or damage to training equipment were noted during this period, demonstrating the feasibility of keeping the trainer cart in place. After the initial pilot phase, only 15 additional uses were recorded during the remainder of the study period from June 2024 to March 2025, which represented an average of one to two uses per month.

**Table 1:** Pilot program cart use. PGY: post-graduate year

Descriptive variables	Number	Total
Level of training of participant		
PGY1	9	21 participants
PGY2	5	
PGY3	7	
PGY4	0	
Other	0	
Gender		
Woman	13	21 participants
Man	8	
Logged role during cart use		
Learner	14	21 participants
Instructor	7	
Number of uses logged per participant		
1 use	21	26 times accessed
>1 use	5	

Survey results

There was less survey participation than anticipated, with few post-survey responses. Eight participants completed the pre-survey. Most participants (62.5%) reported using the task trainers with another person, most often with a near-peer, for example, a PGY1 resident using with a PGY2 resident or a PGY2 using with a PGY3. Self-reported pre-intervention confidence levels correlated with the number of prior procedures reported, with participants who reported a greater number of prior procedures rating their pre-intervention confidence as very high (Pearson's correlation=0.78). Fifty percent of participants reported using the cart for less than 5 min, while 50% reported using the cart for 15-30 min, indicating a dichotomy in the type of use. Four participants completed post-intervention surveys (Table 2).

**Table 2:** Pre- vs. post-survey confidence. CVC: central venous catheter; ETI: endotracheal intubation

Participant ID	Pre-CVC	Post-CVC	Change	Pre-ETI	Post-ETI	Change
1	5	5	-	5	5	-
2	5	5	-	3	4	+1
5	4	4	-	4	4	-
12	5	5	-	5	5	-

Although participants reported varied uses of the cart, including solo practice and rapid-cycle deliberate practice with a coach, no survey participants reported using the cart for just-in-time (JIT) training or for objective skill review (e.g., referencing the mastery checklist in the cart or reviewing self-filmed performance). Participants who completed the post-survey offered free-text responses around their experience with the trainer cart (Table 3).

**Table 3:** Post-survey free text responses.

What did you like?	What would you improve?
Convenient to use during downtime on shift	Increased variety of procedure trainers
Ability to perform real-time teaching in the clinical environment	Change the airway manikin to the lighter/smaller head for ease of portability and access, add a few laminated "best practice" sheets for easy reference, such as difficult airway card or central line checklist, or even a couple of laminated anatomic diagrams with real pictures
So great having them available	Get rid of the lockable curtain in front of the cart. Laminated sheet with bold content list on bins, smaller airway trainer (just head and posterior pharynx as opposed to lungs too)
Located in the department - easy to pull out and use for practice on a slow morning	Greater organization of equipment. Removing excess redundant equipment. Adding relevant equipment (e.g., a rigid stylet - I requested one from central supply, and it is now in the airway trainer box)

Interview results

Of the 27 EM residents invited to participate, seven (26%) completed interviews. Participants represented post-graduate year (PGY)2-PGY4 levels in a four-year urban academic EM program, with near equal gender distribution. The following three main themes emerged: practice makes perfect, applications beyond the tool, and limitations, barriers, and solutions.

Practice makes perfect

Residents emphasized that the value of task trainers lies in deliberate practice. Repetition was described as increasing comfort, confidence, and procedural readiness in a psychologically safe environment. Even though trainers cannot fully replicate patient haptics, fidelity was considered sufficient to reinforce muscle memory, familiarize learners with kits, and reduce anxiety around rare procedures, as reflected in their experiences: “Always the more you practice, the better you're going to do clinically.” - participant one. “You can watch videos, but the muscle memory is very important.” - participant seven. “Just knowing what's in the kit as opposed to having to figure that out, plus the procedural aspect in real time, was helpful.” - participant three.

Applications beyond the tool

Residents preferred practicing with peers rather than alone, often making practice interactive and engaging. Task trainers supported just-in-time teaching, refresher training, and resource stewardship by preserving clinical supplies. Several participants described direct transfer to patient care as follows: “I used it both to familiarize myself and to teach one of the interns before she needed to do a chest tube on her patient. It improved her success rate.” - participant two. “I had a patient who had a severe bleed going on in the oropharynx, and we were trying to do a scope on the patient. I think the fact that we were able to try it, then do the sim, and then try it again was helpful. And then I actually had much more comfort using the tool when I'm in ICU right now, when we're bronching someone.” - participant three.

Participants also anticipated that task trainers would play an important role in supporting lifelong learning and continuing medical education (CME), as reflected in the following perspectives: “I think first and foremost, I want to take good care of patients, right? To provide good clinical care, I want to obviously be facile with these procedures, especially if I'm going to be the one teaching and supervising. You really want to have that kind of familiarity with the procedure to be able to teach.” - participant five. “I think it's going to be very important for CME. I'm going to be supervising residents (and) my skills are going to decay quickly if I don't actually practice.” - participant seven.

Limitations, barriers, and solutions

The most cited challenge was limited time and competing clinical demands. Some noted that the cart was “out of sight, out of mind,” or difficult to access. Others found fidelity inadequate for advanced learners, who valued trainers more for unfamiliar procedures than routine ones. “When I'm in the ED, I'm clinically working 99% of the time; it's hard to have the time because you have all your other clinical tasks on shift.” - participant one. “It feels quite different than how it feels doing it on a real person. So that was a little bit frustrating. So I was like, yes, I understand this kit and the mechanics, and I can do this on this little mannequin. But I do the same thing to a person, and I feel like it doesn't work.” - participant six.

Suggested improvements included increasing the cart's visibility and accessibility, embedding it in the curriculum, and gaining stronger buy-in from attending staff to normalize its use during shifts. “Every single time I've used a task trainer on shift, it can happen very quickly, and it's very fun to use, so I think it's an accessibility thing.” - participant seven. “Having attended buy-in is really helpful; if they're okay with us taking 10 min and just stepping away, that is helpful.” - participant three. Table 4 summarizes a full list of codes, definitions, and excerpts from transcripts.

**Table 4:** Qualitative analysis codebook. TT: task trainer; CME: continuing medical education

Themes	Codes	Definition	Example
Practice makes perfect	Deliberate practice	Intentional practice on a specific skill, often with feedback and/or coaching for the purpose of improvement (active, repetition, refreshing)	“I think any time you can do a sim, it helps. You know, one time, a thousand times, it still helps. You can learn something every time.” - participant two
	Safe practice	Minimum patient harm/minimum self-harm	“Actually, having like a safe space to practice that was lower stakes was pretty helpful.” - participant four
	Competence	The ability to perform the procedures correctly	“I definitely felt more comfortable (after practicing with the cart). I mean, I don't think I was more competent. I think that it just still needs more experience. But I think I was more comfortable at least knowing you know how to look at the screen and know what’s what, especially with the orientation and how to actually use my wrist for manipulation" - participant three
	Confidence	Feeling of being able to perform procedure successfully/correctly (comfort)	"The more repetition that I was getting, the more comfortable that I felt doing whatever the procedure was.” - participant one
	Muscle memory	Ability to reproduce a particular movement without conscious thought	“You can watch videos, you can recite everything in your head, but, like, the muscle memory is very important for learning these specific skills.” - participant seven
	Fidelity	Even though not "real" it has real learning applications	"I find those task trainers really good, for the most part, in terms of being able to use the equipment in a realistic way. Obviously, I don't think it feels the same exactly. I think plastic is a little bit more rigid than what a human feels like… But I think it's really great for your hand-eye coordination when it comes to getting your reps in for that.” - participant seven
	Accessibility	The TT is physically available, it's easy to get to, it's free to be used, and can practice high acuity low occurance (HALO) procedures	"If you don't have that spaced repetition and if you don't do it frequently enough, that's something that's easily forgotten in the heat of the moment.” - participant five
	Decreased extraneous cognitive load	Anything and everything that distracts or lessens the benefit of TT sims (being stressed, not buying in, lack of fidelity and/or realism, worried about breaking the TT, time restraints, negative emotions you carry into the TT sim)	"Just knowing what's in the kit as opposed to having to figure that out plus the procedural aspect in real time, was helpful." - participant three "I would say the more practice the better and less stressful in the moment, that is, when you're clinically needing to do that procedure or the skill.” - participant one
Beyond the tool - applications in the learning environment	Just-in-time training	Skill practice just before the need to perform it	“I used it both to familiarize myself and to teach one of the interns before she needed to do a chest tube on her patient. And so, we went through it to remind myself of what to do, but also to teach at the same time and show her what we were doing. I kind of worked through my own memory blanks before we went in there. It was a refresher. And then I think it allowed her to touch and feel and practice before we actually went in to do it, and I think it improved her success rate.” - participant two
	Lifelong learning	Continuing practice (self-initiated, refresh, maintain skills, room for personal improvement)	“I think it's going to be very important for CME. It really depends on the site I work at, you know, if I'm going to be working at supervising residents. My skills are going to decay quickly if I don't actually practice.” - participant seven
	Supports active learning	Different factors that contribute to supporting learning through TT sim (train with a partner, quiet place, choosing the right time, right head space, fun/positive emotional experience)	I think doing it by myself is fine, but I also feel like, if I'm doing a procedure by myself, it's like I don't know what I don't know.” - participant three
	Teaching tool	The TT is not just for my learning, but as a teaching tool too	“And X made a little game out of it. So, X put some little pieces in the simulated airway inside the mannequin, and we had to find those. So, it was fun.” – participant three
	Resource stewardship	Saving cost from opening multiple kits for training/teaching purposes	“I like having access to it. I like that I don't have to open a new kit”- participant two
	Transfer to practice	How TT informs clinical practice or the approach to real practice (stress, skill, etc.)	“I had a patient who had a severe bleed going on in the oropharynx, and we were trying to do a scope on the patient, but it was really hard to just take a look there. And so (attending Z) brought one of the mannequin heads and we just practiced doing it in there. I think the fact that we were able to try it, do the sim, and then try it again to make sure that we were able to successfully do it was helpful. And then I actually had much more comfort using the tool when I'm in ICU right now, when we're bronching someone.” - participant three
Limitations, barriers, and solutions	Lack of realism	Doesn't feel real (rough, stiff, fragile)	“I don't really like that much because they don't feel very accurate at all.” - participant six
	Poor accessibility/availability	Not easy to get to physically	"Just seems really complicated to figure out how to use it" - participant two “I remember on my teaching block, I wanted to use it. But then I couldn't find it. which is sad because I would love to use it." - participant seven
	Not one size fits all	TT works for some, not all procedures/some better than others	“The material of the mannequin changes in its importance, depending on what skill you're trying to practice” - participant two “I think the procedures with which I have more comfort, I found that task trainer is at this point maybe less useful" - participant five “I still find that like LP models aren't as helpful in real life because like they're always too perfect. You're walking through the steps, but it doesn't really help troubleshooting." - participant seven
	Poor fidelity	The degree of exactness with which the TT experiences reality (environment, manikin, scenario, team, etc.)	“I really don't think that the way I intubate on an airway trainer is the same as how I do it on a person.” - participant six
	Room for TT improvements	Suggestions for improving the experience	“Having attending buy-in is really helpful” - participant three “I think it has to be worked somewhat into the curriculum.” - participant seven "Every single time I've used a task trainer on shift, it can happen very quickly, and it's very fun to use, so I think it's an accessibility thing.” - participant seven
	Lack of support/bandwidth	Different factors that prevent/discourage learning through TT sim (lack of free time during shifts, competing priorities/patient care demands, lack of supervisor support to use TT)	“When I'm in the ED, then I'm like clinically working 99% of the time. So, it's hard to have the extra awareness to be like, oh, there's something like additional non-clinical learning I could be doing, or usually I just don't have time because you have all your other clinical tasks on the shift.” - participant one “It seems like a little bit out of sight, out of mind if I don't like actively think about it, I don't actually run into it day to day, I just kind of forget about it sometimes.” - participant four

## Discussion

ED clinicians were eager to interact with the task trainers when they were a novelty in the department, but participation fell off over time. Lack of advertising, low visibility of the cart’s location, difficulty unlocking the cart, and difficulty using bulkier trainers were some of the barriers described. Additionally, trainees’ competing clinical priorities resulted in a perceived lack of time, bandwidth, and a sense of cultural taboo around seeking out trainers while the department felt busy. This aligns with a prior study of JIT procedure guides, which found that EM physicians reported limited time as one of the most common barriers to JIT training [17]. Mitigating these barriers will be essential to successful future implementation. Rotating different procedure training supplies through the cart at regular intervals (e.g., every three months) may keep the experience fresh for repeat users and create opportunities for spaced repetition when removed supplies are cycled back through the cart. Swapping out the larger, higher-fidelity task trainers for smaller, slightly lower-fidelity task trainers could increase ease and speed of use on shift. The portability and immediate accessibility of digitally accessible images, videos, algorithms, and guides may be more useful for JIT review in a truly emergent situation, while task trainer practice may be better suited when several minutes can be spent preparing for the procedure. Having both task trainers and quickly accessible visual aids should be available for use in appropriate scenarios. Incorporating video review to provide more objective feedback on performance may make future iterations of this cart more effective for learners. Having a well-defined and visible location of the cart and expectations around when and how it should be used (e.g., during a senior resident's teaching rotation) may further reduce some barriers to access.

Resident interviews highlighted the motivations and goals of using task trainers - getting “reps” and building muscle memory in a safe setting with feedback and coaching to support growth. The centrality of deliberate practice in EM resident procedural training aligns with prior evidence from other medical specialties and adds the unique perspective of EM trainees [18-21]. Importantly, nearly all trainees interviewed described a role for task trainers after the completion of residency training. Ongoing and regular practice with task trainers to minimize procedural anxiety, improve patient care, and improve the quality of teaching provided to future trainees was all described, and having equipment conveniently available in the clinical space could support this.

Limitations

This study had several limitations. It was conducted at a single site with a small number of participants, and the survey portion was underpowered. Voluntary participation likely led to selection bias; those who chose to participate likely had more positive perceptions of simulation-based learning compared to non-participants. Although interview participants described instances in which their practice with task trainers directly applied to their clinical behavior, there is potential for recall bias. Additionally, the interviewer was known to the participants. All interviews were prefaced with language intended to address the reflexivity of both parties and to allow for a psychologically safe and open dialogue.

This study did not use an objective assessment of skills to evaluate change after using the trainer cart, and confidence levels have been described as an imperfect measure of competence [22]. Future studies should be designed to assess changes in skill competency through checklists, global rating scales, or other objective and validated measures.

Although this study aimed to understand the role of task trainers in EM resident education, which may indirectly affect patient care, it did not examine patient-centered outcomes. Review of the electronic medical record (EMR) data, looking for a change in rates of procedures performed or complication rates before vs. after implementation of the intervention, was considered but determined not to be meaningful for the purposes of this exploratory investigation with a small sample size and several confounding variables due to concurrent changes in department flow and resident curriculum.

Future directions

The most immediately actionable implications of this study are the changes that can be undertaken to improve future iterations of a similar trainer cart. In situ task trainers should be made visible, accessible, and incorporated into formal curricula or departmental teaching culture. They can be utilized to enhance on-shift teaching via deliberate practice or just-in-time training in pairs or groups; although solo practice was seen in this study, trainees described receiving feedback and coaching as the biggest value-add of this intervention, and educators should ensure a mechanism for this. Additional studies involving additional training programs and larger study populations will be needed to produce more generalizable results. Future studies should aim to incorporate objective measures of change in procedural skills and consider using additional data sources, such as EMR data, to triangulate and better understand the impact of ED-based task trainers on procedural skill acquisition and retention. Additionally, because the cart is accessible to all role groups in the ED, additional areas of future research could assess how these supplies support skills maintenance in other role groups, such as EM attending physicians, or support interprofessional education in the department.

## Conclusions

Making simulation-based education more conveniently accessible and closer to real-world practice by placing a task trainer cart in the clinical setting was well-received by the clinicians who used it. Although this study was limited by few survey responses and lack of objective skills assessment, the insights shared by EM residents during semi-structured interviews provided better understanding into the ways that the task trainer cart and similar interventions support deliberate practice, increase confidence and competence, are applied to patient care, and fit into a longitudinal learning curve of procedural skill acquisition and maintenance in EM trainees.

## Figures and Tables

**Figure 2:**
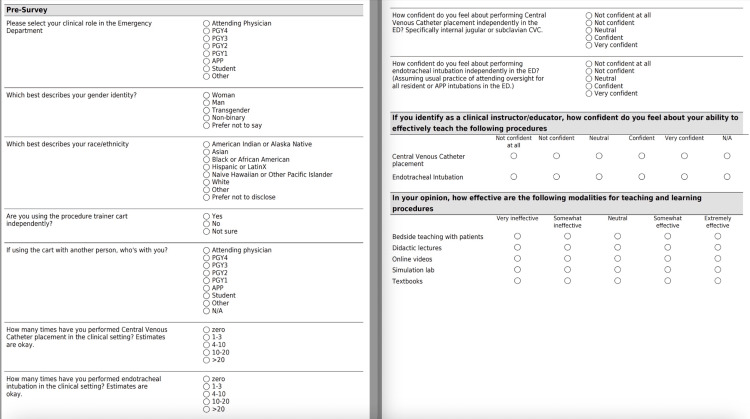
Pre-intervention survey. CVC: central venous catheter; PGY: post-graduate year; APP: advanced practice provider

**Figure 3:**
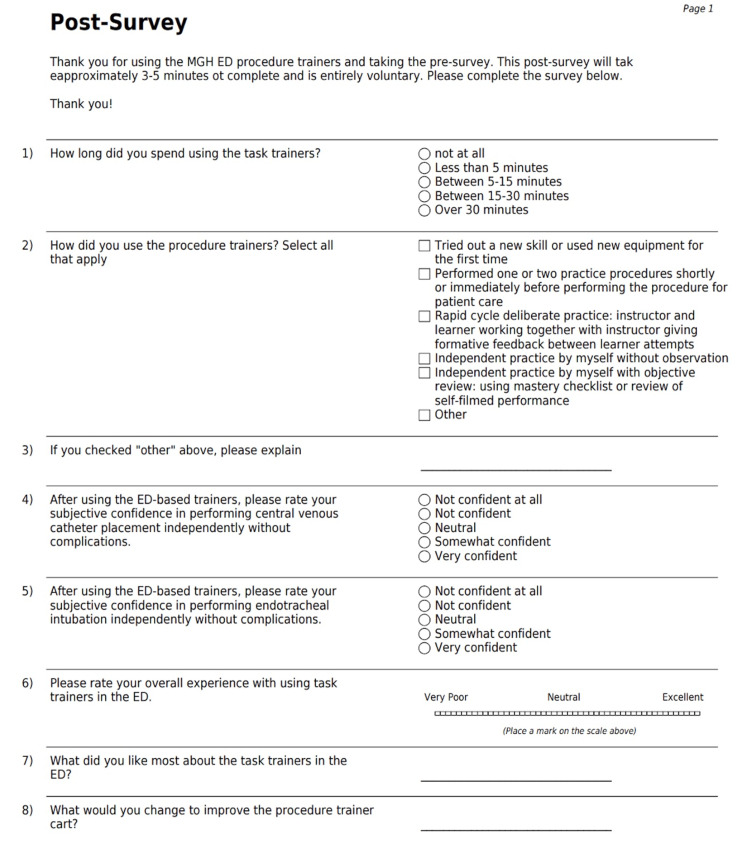
Post-intervention survey.

**Figure 4:**
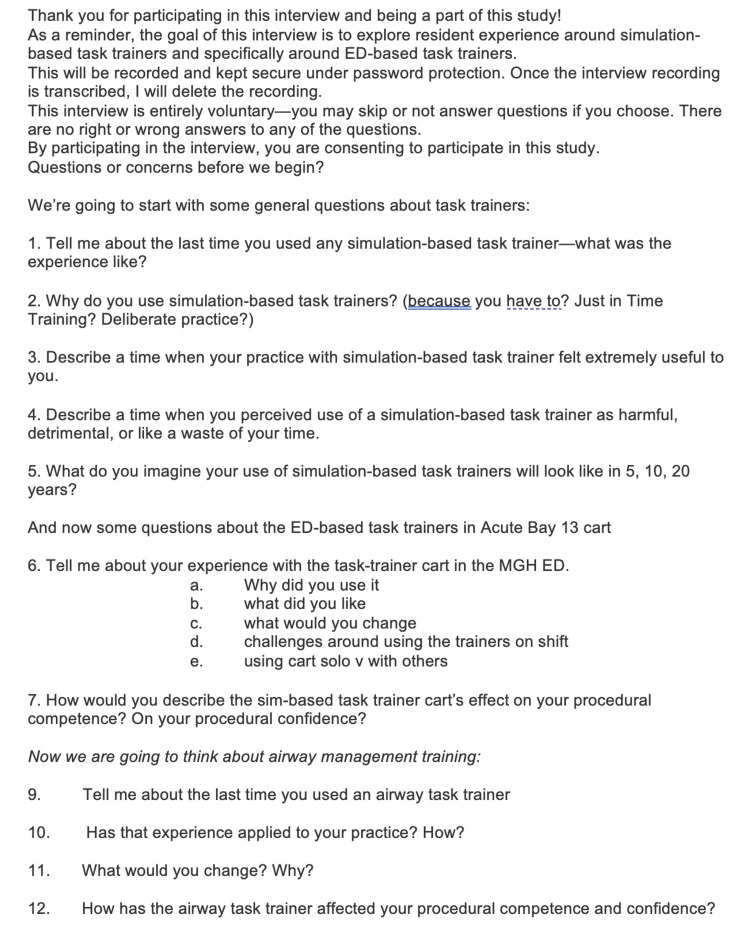
Semi-structured interview guide.

## Appendices

Appendix 1

Surveys were administered before (Figure 2) and after (Figure 3) participants used the task trainer cart. 

Appendix 2
